# Chloroplast Biogenesis-Associated Nuclear Genes: Control by Plastid Signals Evolved Prior to Their Regulation as Part of Photomorphogenesis

**DOI:** 10.3389/fpls.2015.01078

**Published:** 2015-12-10

**Authors:** Alison C. Hills, Safina Khan, Enrique López-Juez

**Affiliations:** School of Biological Sciences, Royal Holloway University of LondonEgham, UK

**Keywords:** chloroplast development, photomorphogenesis, plastid signals, LHCB, DET1, COP1 *Arabidopsis*, GLK, gymnosperm photomorphogenesis

## Abstract

The assembly of photosynthetically competent chloroplasts occurs in angiosperm seedlings when first exposed to light, and is due to the control by light of photosynthesis-associated nuclear genes (PhANGs), also dependent upon plastid-to-nucleus “biogenic” communication signals. The relationship between light- and plastid signal-regulation of PhANGs is close but poorly understood. In contrast, many conifers green in the dark and the promoter of a pine PhANG, *Lhcb*, is active in the dark in tobacco. Here, we show that the activity of this promoter in tobacco is sensitive to plastid photobleaching, or to the inhibition of plastid translation in the light or the dark, and the same interventions reduce expression of the native gene in pine seedlings, demonstrating classic plastid biogenic signaling in gymnosperms. Furthermore, *Arabidopsis* mutations causing defective plastid biogenesis suppress the effect in darkness of mutations in COP1 and DET1, repressors of photomorphogenesis, for the expression of several PhANGs but not a photosynthesis-unrelated, light-regulated gene. GLK transcriptional regulators mediate the response of *LHCB* but not of other tested PhANGs. We propose the ability to suppress PhANG response to positive plastid biogenic signals in the dark may have contributed to the evolution of light-controlled chloroplast biogenesis.

## Introduction

The development of flowering plant chloroplasts occurs in the light, and involves the expression of 1000s of genes encoded in the nucleus, the import of their products into developing plastids, as well as the expression of ca. 120 protein and RNA-encoding genes by the genome of the chloroplast itself (Waters and Langdale, 2009; Jarvis and López-Juez, 2013). Light is a key inductive signal for the expression of genes involved in the assembly of a photosynthetically competent chloroplast, the so-called photosynthesis associated nuclear genes (PhANGs). In seedlings germinated in the absence of light leaf development is repressed and plastids in the cotyledons develop as etioplasts, containing partially developed internal membranes and a chlorophyll precursor, protochlorophyllide, associated to a light-requiring protochlorophyllide oxido-reductase (Reinbothe et al., 1996). This renders seedlings photosynthetically incompetent. At the same time the expression of PhANGs in cells developing chloroplasts is closely coordinated with the functional state of the plastid. If ongoing plastid biogenesis is impaired, by failure to safely complete chlorophyll biosynthesis (because of photooxidative damage to membrane complexes, caused by carotenoid synthesis mutations or chemical inhibitors like norflurazon), or to express the chloroplast genome (because of organelle translation mutations or inhibitors), or to import nuclear-encoded proteins, PhANG expression is also down-regulated (Inaba et al., 2011; Chi et al., 2013). This reveals the existence of plastid-to-nucleus communication, also called plastid retrograde signaling, more specifically plastid biogenic signaling. The term “biogenic” is used to distinguish it from operational or environmental signaling, which refers to the later influence of functional but stressed chloroplasts on nuclear genes when subjected to environmental challenges (Woodson and Chory, 2012; Pogson et al., 2015). Biogenic signals resulting at least from defects in tetrapyrrole (primarily chlorophyll) biosynthesis and from deficiencies of organellar gene expression can themselves also be, to a degree, genetically separated (Vinti et al., 2000; Mochizuki et al., 2001; Gray et al., 2003; Chi et al., 2013).

Light control of PhANG expression is part of a broader program of control by light of development overall, the so-called photomorphogenesis program, initiated by the activation of phytochrome and cryptochrome photoreceptors. This program contrasts with that of development in the dark, skotomorphogenesis, which instead ensures investment into elongating organs and prevents the development of photosynthesising leaves (Arsovski et al., 2012). The result is a vast reprogramming of the transcriptome of seedlings when first exposed to light (Jiao et al., 2007). Plastid biogenic signaling, on the other hand, probably involves the production or regulated export of one or more tetrapyrrole signals (Terry and Smith, 2013), and the monitoring of the activity of the chloroplast transcription/translation machinery, involving the plastidic GUN1 protein (Koussevitzky et al., 2007; Woodson and Chory, 2008). In spite of their apparent dissimilarity, multiple observations have highlighted a close relationship between light and chloroplast biogenic control of PhANGs. For example promoter truncations could not remove one response without the other, and plastid development defects led to reduced light responsiveness (Simpson et al., 1985; Bolle et al., 1996; McCormac et al., 2001; Vinti et al., 2005; Ruckle et al., 2012). Even gain of function experiments using light-regulated pairs of promoter elements (Puente et al., 1996; Martínez-Hernández et al., 2002) recreate the light response and the plastid dependence simultaneously.

Such evidence raises the intriguing possibility of light and plastid-to-nucleus signaling to PhANGs sharing mechanisms, even of one potentially being based on the other. Plastid-to-nucleus biogenic signals have been revealed in flowering plants, with their nature and very existence in the green alga *Chlamydomonas reinhardtii* being the subject of mixed evidence (Johanningmeier and Howell, 1984; Johanningmeier, 1988; Ramundo et al., 2013). Meanwhile the presence of phytochrome and cryptochrome photoreceptors has been shown in all major groups of land plants (Sharrock and Mathews, 2006) but the nature of photomorphogenic responses varies. Many gymnosperm seedlings grow partially skotomorphogenically but green in the dark (Alosi et al., 1990; Yamamoto et al., 1991; Burgin et al., 1999). This stems from the presence of a light-independent protochlorophyllide oxido-reductase (Forreiter and Apel, 1993), and from the expression of PhANGs in the dark (Yamamoto et al., 1991; Peer et al., 1996). In fact the promoter of the *Lhcb6* (*cab-6*) gene from black pine, encoding a form of the apoprotein of the major light harvesting complex of photosystem II, is able to drive the expression of a beta-glucoronidase (GUS) reporter gene constitutively in the dark in an angiosperm, tobacco (Kojima et al., 1994). This observation provided a tool which could now be used to analyze the relationship between light and plastid-to-nucleus biogenic signals in the expression of PhANGs.

A central theme in photomorphogenesis is the activation of light-induced genes by transcription factors (notably HY5) whose accumulation is prevented in the dark by proteosomal degradation, triggered by the activity of ubiquitin ligase and associated proteins of the DET/COP class; those proteins therefore act as repressors of photomorphogenesis. The activity of these repressors is abolished in the light by photoreceptor activation (Jiao et al., 2007; Lau and Deng, 2012). Nuclear events of plastid-to-nucleus biogenic signaling are less clear, with a negative role for the ABI4 transcription factor, acting downstream of chloroplast-localized GUN1, having been shown for a subset of genes (Koussevitzky et al., 2007); furthermore the GOLDEN2 LIKE (GLK) family of transcription factors has been demonstrated to play a conserved, positive role in the expression of selected PhANGs across land plants (Fitter et al., 2002; Yasumura et al., 2005; Waters et al., 2009), and to also be involved in the response to defective plastid protein import on such genes (Kakizaki et al., 2009).

In this study we have asked the following questions: is the pine *Lhcb* promoter truly phytochrome-independent in tobacco? Is it dependent on plastid-to-nucleus biogenic signals and, if it is, does this reflect the situation in the original cellular environment in pine? Is there, in *Arabidopsis*, a genetic interaction in the dark between plastid biogenesis and repressors of photomorphogenesis? And what role do GLK transcription factors play in the response to light and plastid-to-nucleus communication? Our results lead us to propose a model in which the photoreceptor control of chloroplast development evolved, in part at least, through the recruitment of DET/COP repressors to suppress to ability of PhANGs to respond to plastid-to-nucleus signals of a positive nature in the dark.

## Materials and Methods

### Plant Material, Light, and Growth Conditions

**FIGURE 1:**
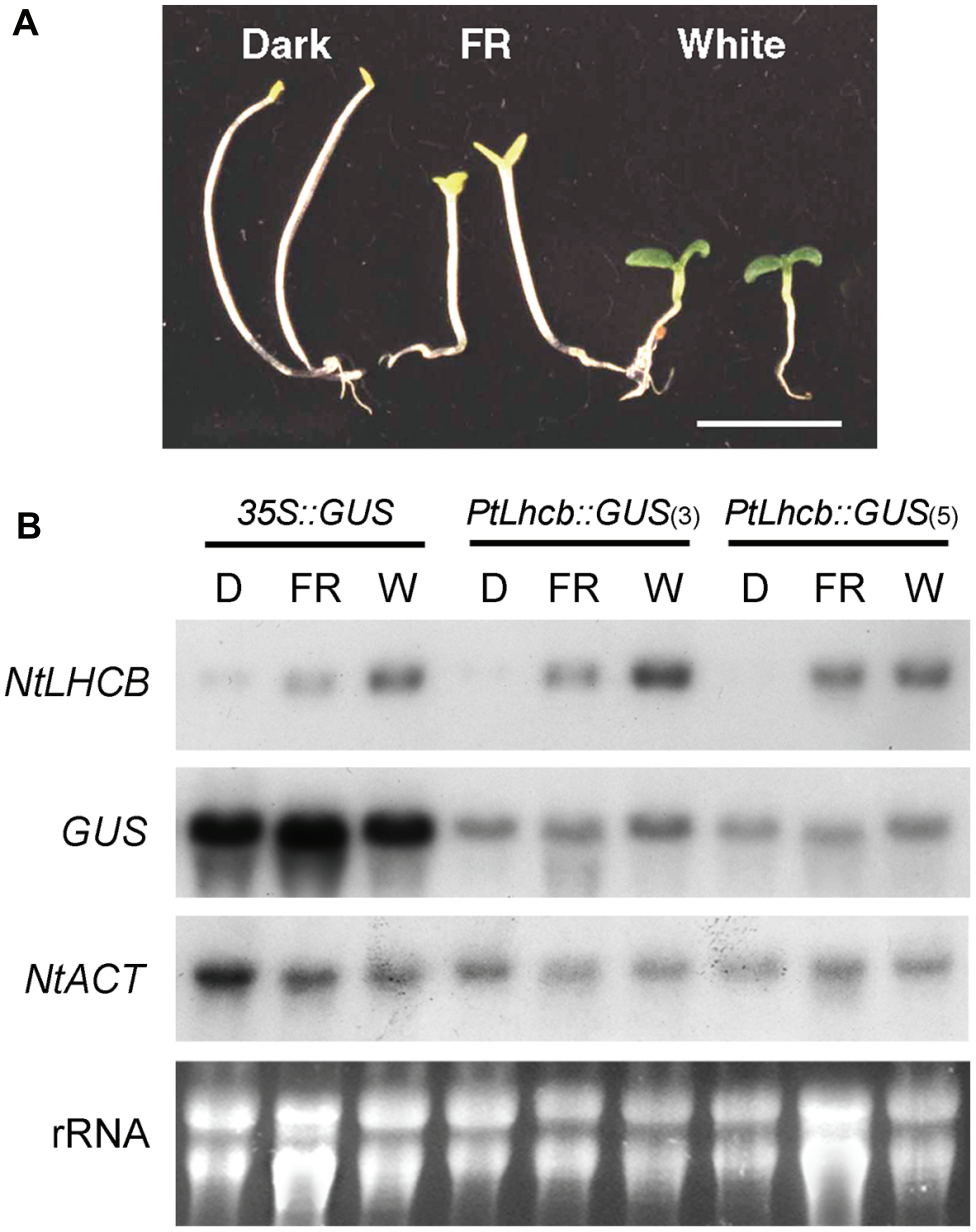
**Morphology and RNA gel blot analysis showing the response of the pine *Lhcb* promoter to light in tobacco seedlings.** **(A)** Tobacco seedlings incubated for 7 days in the dark (D), under continuous far-red (FR) light, or continuous white (W) light, before tissue harvest. The seedlings shown correspond to those of the control, *35S::GUS* line below, but all lines responded equally. **(B)** RNA gel blots of tobacco seedlings containing the GUS reporter under the control of the pine *Lhcb6* (*PtLhcb*) promoter (two independent lines, numbers 3 and 5) or the control, constitutive 35S promoter, and treated as indicated in **(A)** before RNA isolation and fractionation. Blots were hybridized with probes indicated on the left, against the endogenous, tobacco *LHCB7* (*NtLHCB*), the *GUS* reporter, or the tobacco *ACTIN9* gene (*NtACT*) as a loading control. Ethidium bromide-stained total, primarily ribosomal RNA (rRNA) is also shown.

**FIGURE 2:**
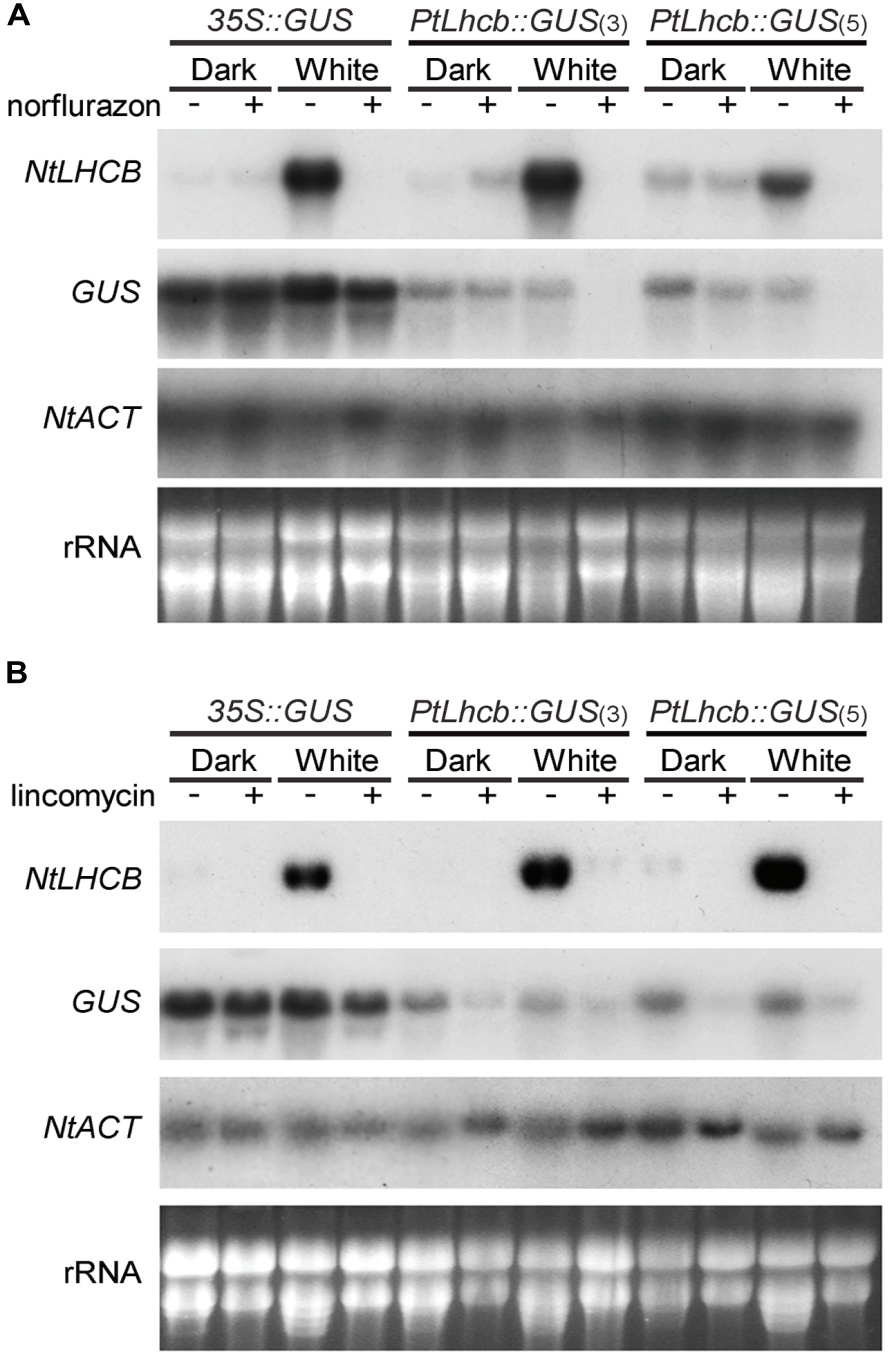
**RNA gel blot analysis showing the response of the pine *Lhcb* promoter to treatments affecting plastid viability in tobacco seedlings.** **(A)** Tobacco seedlings incubated for 7 days in continuous darkness or white light in the presence (+) or absence (-) of the carotenoid biosynthesis inhibitor norflurazon, before RNA isolation and gel blot analysis. **(B)** Tobacco seedlings incubated for 36 h in the dark or white light, followed by 5 and a half days in the presence (+) or absence (-) of the prokaryotic translation inhibitor lincomycin, before RNA isolation and analysis (as in **Figure 1**).

**FIGURE 3:**
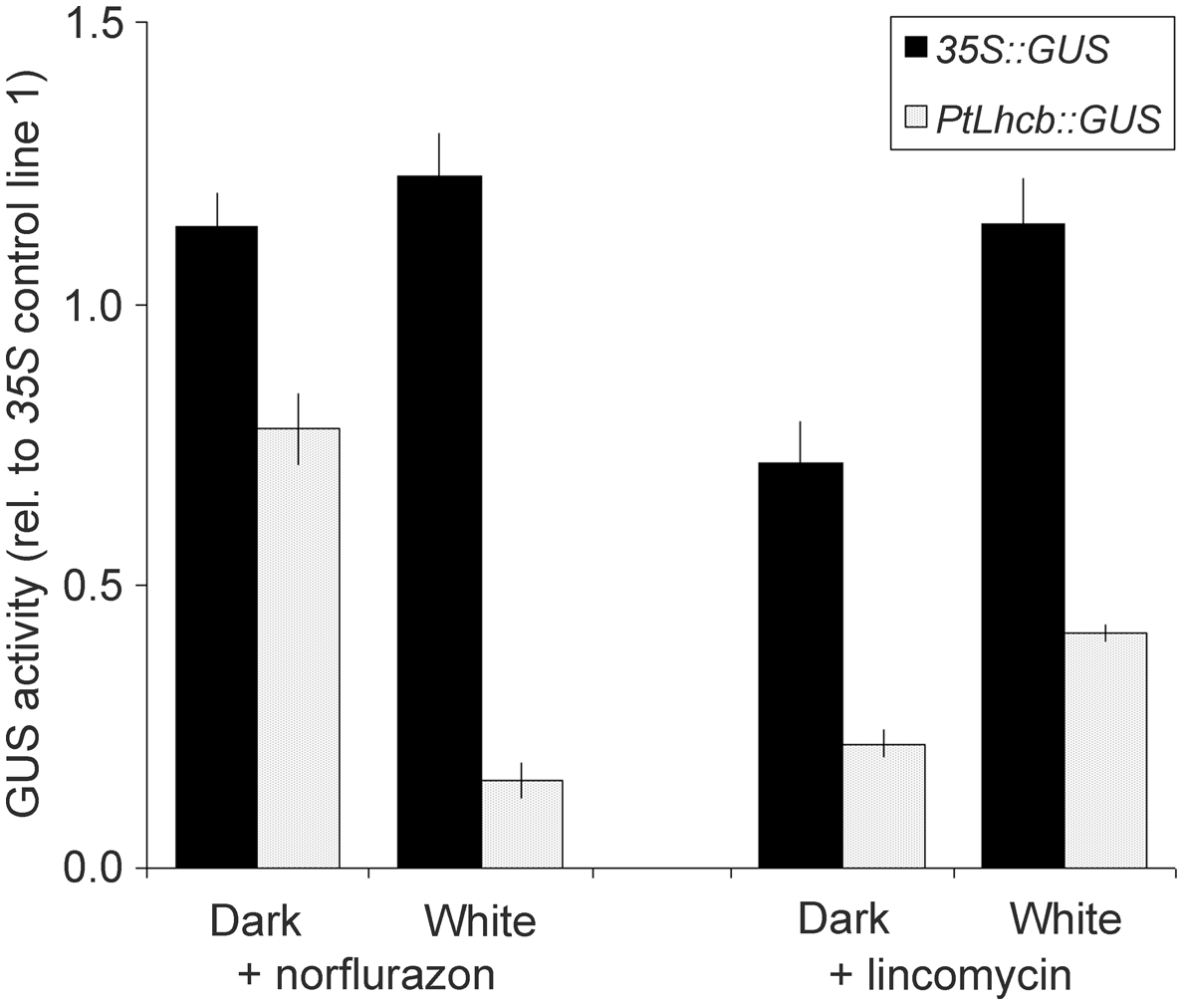
**Glucoronidase (GUS) activity measurements reporting the activity of the pine *Lhcb* promoter in response to treatments affecting plastid viability in tobacco seedlings.** GUS activities, from *PtLhcb:GUS* (line number 5), or *35S:GUS* (line number 18) exposed to treatments identical to those for **Figure 2**, are expressed relative to the activity under the same treatment for a second 35S control line, number 1, in order to compensate for developmental effects (a value of GUS of 1 indicates a response identical to that in control line number 1). Error bars indicated standard error of the mean.

Tobacco (*Nicotiana tabacum* cv. Petit Havana) lines (numbers 3 and 5) carrying 1900 bp upstream of the start codon of the pine *Lhcb6* (*cab-6*) gene driving the GUS-coding sequence, or the *35S* promoter GUS fusion gene (line 18 for experiments in **Figures 1** and **2**, or lines 1 and 18 for **Figure 3**), were described previously (Kojima et al., 1994). Unless otherwise stated, seeds were surface-sterilized, plated on agar-solidified MS medium containing 1% sucrose, and incubated as previously described for *Arabidopsis* seeds (Vinti et al., 2000). Pine (*Pinus thunbergii*) seeds were obtained from Chiltern seeds, Ulverston, UK. Seeds were stored at 4°C for at least 2 months before use. Seeds were surface-sterilized with a 22% solution of hydrogen peroxide for 30 min, before placing on MS medium in magenta boxes (Sigma Aldrich, Poole, UK). Due to erratic germination and slightly uneven growth rate under the different conditions, seeds were monitored daily for less than 30 s under a green safe light (Vinti et al., 2000) until radicle emergence, and grown subsequently for a period between 14 and 21 days (as determined in preliminary experiments), until they reached a stage comparable to that reached by seedlings in the light at 14 days. *Arabidopsis* seeds of the *det1*-1 mutant (Chory et al., 1989), *cop1*-4 (Deng et al., 1991), *ppi1*-1 (Jarvis et al., 1998), and *glk1 glk2* double mutants (Fitter et al., 2002) were kind gifts from J. Chory (Salk Inst.), J. Sullivan (Queen Mary University of London), P. Jarvis and J. Langdale (both University of Oxford), respectively. Seeds of the *cue8* mutant, and its wild type, the reporter gene-containing pOCA108 line (Bensheim ecotype) were lab stocks (López-Juez et al., 1998). Double mutants were isolated from the respective crosses, selecting in the second generation for a deetiolated phenotype in the dark, followed by a visibly pale (*ppi1*) or slow greening (*cue8*) phenotype upon transfer to soil, or by genotyping assays of *glk1* and *glk2* as described (Fitter et al., 2002). For *det1 ppi1* deetiolated mutants were selected in the F2, and those segregating with a paler phenotype identified in the F3. The segregation ratios of the phenotypes as seedlings were consistent with single or double mutant (triple for *det1 glk1 glk2*) genotypes, although the survival rate of combined mutants was reduced.

The growth temperatures for every experiment were 25°C (for tobacco and pine) and 21°C (for *Arabidopsis*).

### Light and Inhibitor Treatments

FR and white light sources were as previously described (Vinti et al., 2000). The fluence rate of white light was 150 μmol m^-2^ s^-1^, and that of FR 15 μmol m^-2^ s^-1^. Norflurazon, a kind gift of P. Bramley (Royal Holloway, University of London), was incorporated into agar-solidified growth media at a final concentration of 10 μM (tobacco) or 100 μM (pine). Lincomycin (Sigma Aldrich, Poole, UK) was added to growth media at a final concentration of 0.5 mM (tobacco) or 10 mM (pine). For lincomycin treatment, seeds were placed on sterile filter paper laid onto lincomycin-free, agar-solidified medium, and transferred to lincomycin-containing plates after 36 h (tobacco) or on the day of radicle emergence (pine).

### GUS Activity Assays

Glucoronidase activity was measured using a fluorogenic substrate as described (López-Juez et al., 1998). Each experiment used three samples of 5–10 seedlings and all experiments were repeated 2–4 times, with results given being the average of all combined samples.

### RNA Gel Blot Analysis and Quantitation

RNA was extracted as previously described after 7 days (tobacco) or 5 days (*Arabidopsis*) of growth of seedlings in continuous light or continuous darkness (Vinti et al., 2000). Tissue harvest nevertheless took place at the same time of day as the start of growth, to avoid any remaining circadian effects. RNA was extracted from cotyledons of pine seedlings as above, followed by purification through the RNA-binding column of a commercial kit (RNeasy Plant Mini, Qiagen, Manchester, UK). RNA gel blot analysis, hybridisation with ^32^P-labeled probes and quantitation using a phosphorimager and ImageQuant software were carried out as described (Vinti et al., 2000). Probe templates used for hybridisation were produced by PCR-amplification from genomic DNA or plasmids, and are given as (**Supplementary Table S1**).

### Quantitative Real-time RT-PCR

*Arabidopsis* RNA was extracted using the RNeasy Plant Mini kit (see above), quality-checked by agarose gel electrophoresis, and 1 μg aliquots reverse-transcribed using the Quantitec kit (Qiagen). cDNA was used for real-time amplification in a Rotorgene Q (Qiagen) as previously described (López-Juez et al., 2008), except that a JumpStart SYBR^®^ Green Quantitative PCR master mix (Sigma Aldrich) was utilized. Relative quantitation for each target gene used the ΔCt method against the geometric mean of the expression of two constitutive genes, *ACT2* and *UBQ10*, and is presented relative to wild type in the dark. Data for *cop1 cue8* are presented twice, relative to Col-0 (wild type of *cop1*) and to pOCA108 (wild type of *cue8*). Gene identifiers and corresponding primers were as listed (**Supplementary Table S2**).

### Sequence Analysis

Bioinformatic searches of homologs of the *Arabidopsis* DET1 (At4g10180) and COP1 (At2g32950) proteins were carried out in GenBank^1^, among sequenced full or partial genomes of embryophytes and among translated versions of mRNAs and expressed sequenced tags of conifers. Alignment of sequences used Clustal Omega^2^ and the result was displayed using BoxShade^3^.

## Results

### Expression of the Pine *Lhcb* Promoter is Largely Phytochrome-independent in Tobacco

The study by Kojima et al. (1994) described a small degree of light responsiveness of the pine *Lhcb* (*cab-6*) promoter in tobacco seedlings, which could be attributed to developmental effects as it could also be observed in control lines with a constitutive reporter driven by the *35S* promoter. We reanalyzed this issue by exposing the transgenic tobacco seedlings to treatments specifically activating the main deetiolation photoreceptor, phytochrome A, and comparing the expression of the pine *Lhcb*-driven *GUS* reporter with that of the endogenous tobacco *LHCB* gene. We used two independent lines containing the pine *Lhcb::GUS* construct -lines 3 and 5 (Kojima et al., 1994)- and a control line containing *35S::GUS* -line 18 (Kojima et al., 1994)-, and *GUS* and *NtLHCB7* probes. The results show a clear de-etiolation response of the tobacco seedlings under continuous far-red light (FR) treatment (**Figure 1A**), and a very large increase under FR or white light of steady-state mRNA levels of the native *LHCB* genes, but only small (*35S*, *PtLhcb* line 3) or none at all (*PtLhcb* line 5) of those of the *GUS* reporter (**Figure 1B**; for blot quantitation see **Supplementary Figure S1**).

### Expression of the Pine *Lhcb* Promoter is Dependent on Plastid-to-nucleus Biogenic Communication Signals in Tobacco

The lack of phytochrome response of the *PtLhcb6* gene could correlate with a lack of plastid signal response or, alternatively, the responses to plastid and light signals could be different from those of the endogenous tobacco gene. We therefore examined the response of the pine promoter to plastid signals, both those revealed by photooxidative damage that follows carotenoid synthesis inhibition by norflurazon, and those dependent on plastid translation which is blocked by the antibiotic lincomycin (**Figure 2**, with blot quantitation in **Supplementary Figure S1**). Activity of the pine *Lhcb* promoter (represented by steady-state *GUS* mRNA levels) was clearly sensitive to norflurazon treatment in the light (**Figure 2A**). The treatment had negligible effects on pine *Lhcb* promoter activity or tobacco *LHCB* mRNA level in the dark as expected (when carotenoid absence causes no phototoxic damage). Plastid translation-dependent signals were capable of regulating the pine *Lhcb* promoter activity in the light and in the dark (**Figure 2B**). The native *NtLHCB* mRNA decreased in response to lincomycin to a much greater extent than the *PtLhcb*-driven *GUS* mRNA did (**Figure 2B**). This could be evidence that the gymnosperm promoter is less tightly regulated by plastid translation-dependent signals than the angiosperm one is, or it could be explained by additional, post-transcriptional regulatory mechanisms of the angiosperm mRNA.

We confirmed the plastid signal-dependence of the pine *Lhcb* promoter in tobacco by carrying out further seedling GUS activity assays (**Figure 3**). To avoid developmental effects of the treatments on overall protein synthesis capacity (Kojima et al., 1994), activities were expressed relative to those in a separate control 35S line. Again these data showed a very clear decrease in *PtLhcb*-dependent GUS (of around 85%) upon plastid photooxidative damage, and a smaller (60–80%) decrease upon plastid translation inhibition.

### The Expression of the *Lhcb* Gene in Pine is Dependent on Plastid-to-nucleus Communication Signals

**FIGURE 4:**
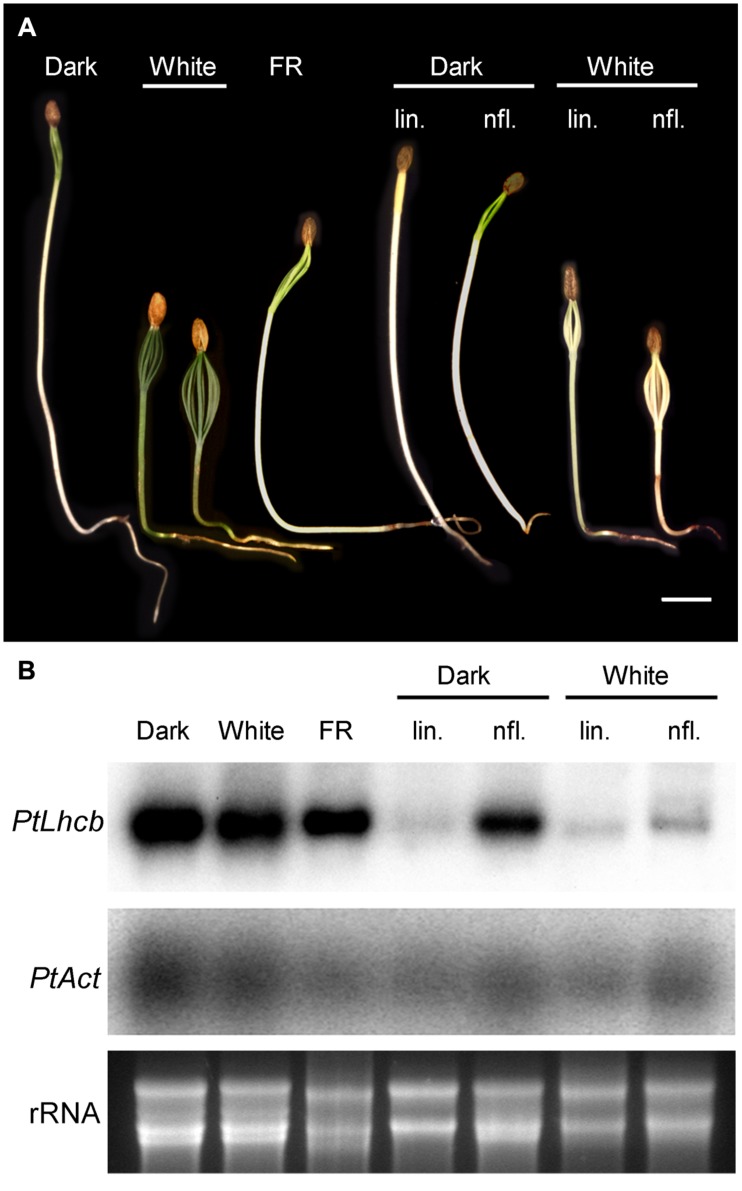
**Response of pine seedlings to light treatments or treatments affecting plastidviability.** **(A)** Photographs of pine seedlings grown in the dark, under continuous far-red (FR) or white light and in the presence or absence of inhibitors (lin: lincomycin; nfl: norflurazon). Seedlings were grown for 14 days from the day of root emergence (under white light), or for a length of time necessary to reach a comparable degree of development of the cotyledons (up to 21 days in the dark). For inhibitor-treated seedlings, seeds were germinated in the absence of inhibitors, and transferred to the presence of inhibitors on the day root emergence was visible. **(B)** RNA gel blot analysis of seedlings treated as shown in **(A)**. *PtLhcb*: pine *Lhcb6* gene. *PtAct*: pine *Actin* gene. Ethidium bromide-stained total, primarily ribosomal RNA (rRNA) is also shown.

The results above prompted us to investigate the existence of plastid-to-nucleus signals in seedlings of pine itself. This was necessary to assess whether the GUS reporter activity in tobacco was an accurate representation of the behavior of the native gymnosperm promoter, as well as to establish whether the signaling machinery is indeed present in pine cells. We examined phytochrome-dependent responses at the same time. These were challenging experiments because of the non-synchronous germination of seeds, and because the inhibitors were only effective at high doses possibly due to inefficient take-up. We monitored the germination of seeds individually through a very brief, safe light exposure, and examined the expression of the PhANG *Lhcb6* relative to that of a constitutive *Act* gene, encoding actin. The results in **Figure 4** confirm the ability of seedlings to green in the absence of light. However, it is clear that other known photomorphogenic responses do take place: seedlings were substantially shorter and cotyledons larger under white light. FR was able to trigger a partial photomorphogenic response. Correspondingly with the green phenotype, no change in the expression of *Lhcb* under light control was apparent. However, the *PtLhcb* expression responded to inhibition of plastid function. Growth of seedlings on norflurazon had a small effect in the dark and caused a very substantial reduction of *Lhcb* mRNA levels in the light, demonstrating that most of the response is due to photooxidative damage. Plastid translation inhibition caused decreases in *Lhcb*, relative to *Act*, in the dark and in the light (**Figure 4**, blot quantitation provided in **Supplementary Figure S2**). This confirms that this gymnosperm *Lhcb* gene is dependent on both of these sources of plastid-to-nucleus biogenic communication signals in its native cellular context.

### Plastid Biogenesis Mutations Suppress the Photosynthesis-associated Nuclear Gene Expression in the Dark Caused by *cop1* and *det1* Mutations

**FIGURE 5:**
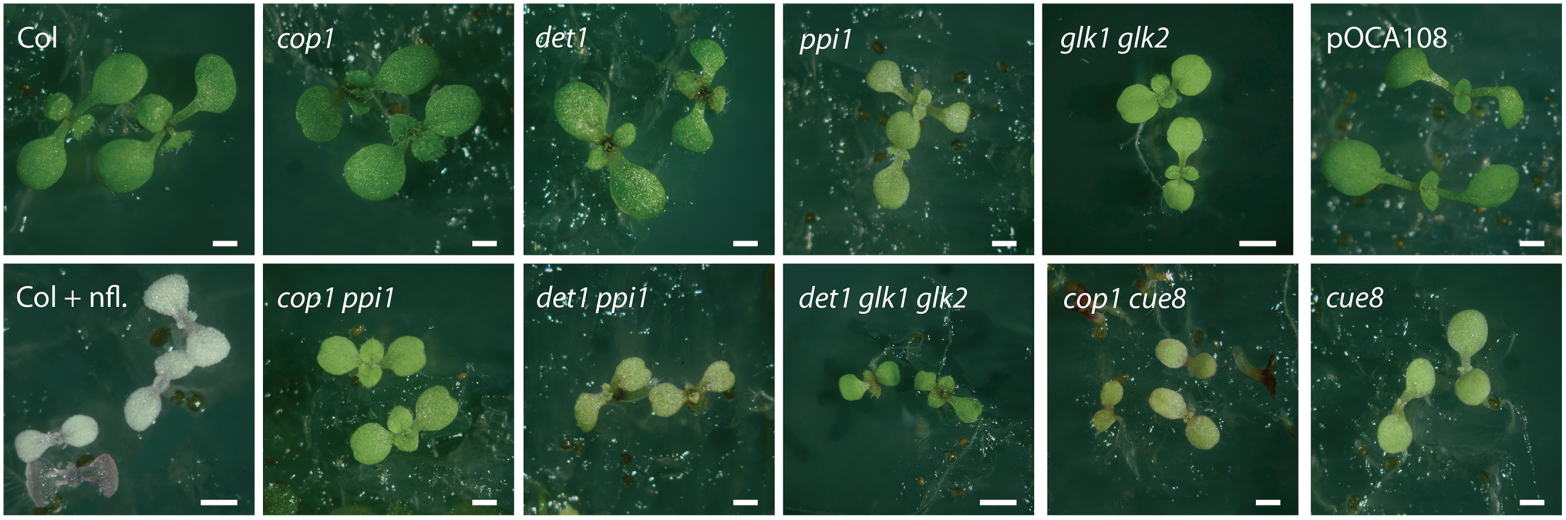
**Images of plastid developmental mutants, photomorphogenic (detiolated) mutants, and mutant combinations.** Seedlings of plastid developmental mutants (*ppi1*, *cue8*, and *glk1 glk2*), de-etiolated mutants (*cop1* and *det1*) and selected mutant combinations were grown in continuous light for 6 days. The wild type for *cop1*, *det1*, *ppi1* and *glk1 glk2* double mutant is Col, the wild type for *cue8* is the line pOCA108, and the double *cop1 cue8* mutant is therefore in a mixed ecotype background. Seedlings of Col grown on norflurazon (nfl.) are also shown. Scale bar: 500 μm.

**FIGURE 6:**
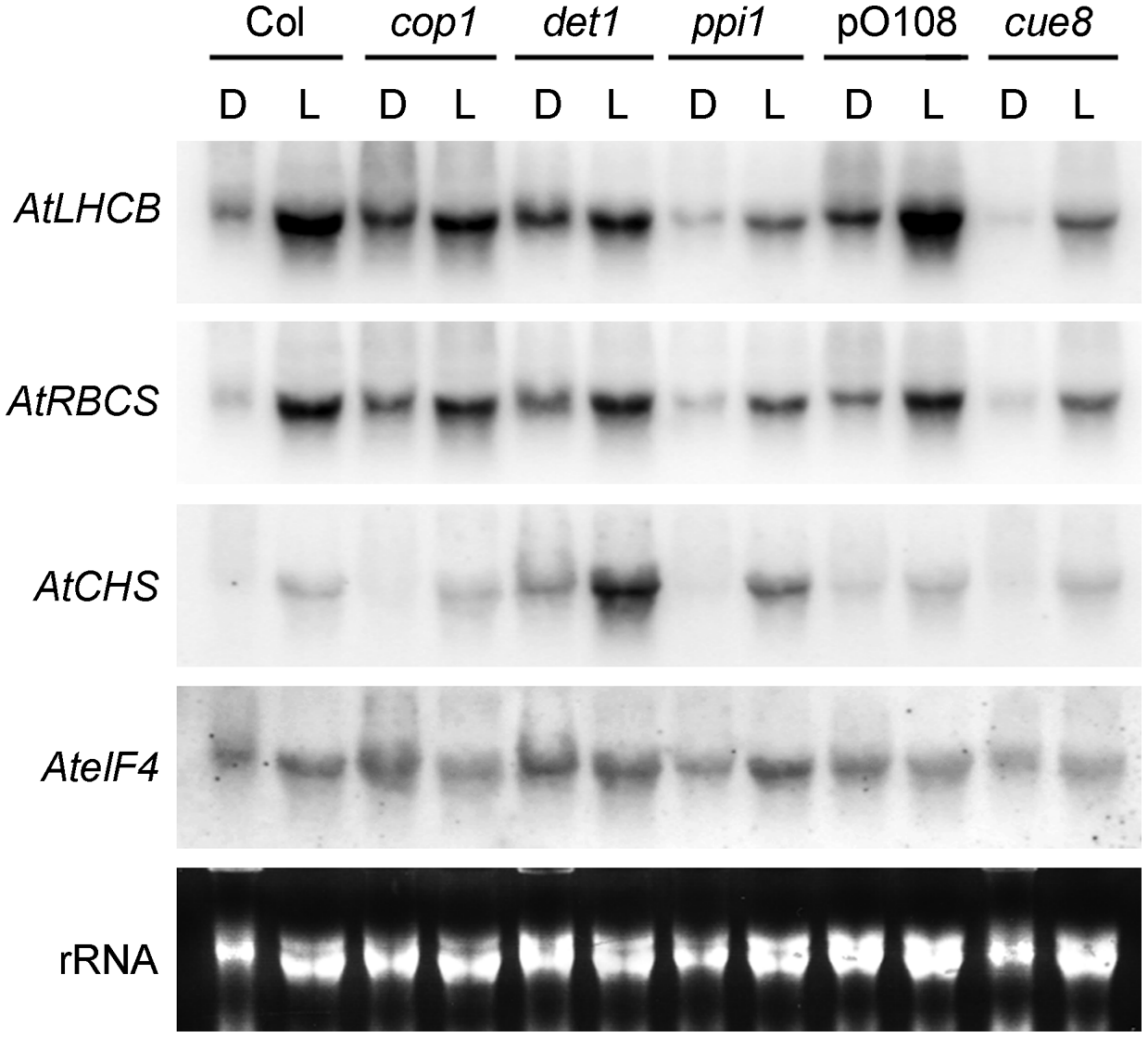
**RNA gel blot analysis showing the response of plastid developmental mutants (*ppi1* and *cue8*) and de-etiolated mutants (*cop1* and *det1*) to light or dark in *Arabidopsis*.** Seedlings of the mutants were incubated in the dark (D) or under white light (L) for 5 days, followed by RNA extraction and gel blot analysis, and compared to their respective wild types grown under the same conditions (Col for *cop1*, *det1* and *ppi1* and pOCA108 for *cue8*). *Arabidopsis* probes used for hybridisation are indicated on the left: these are the genes for LHCB1 (*AtLHCB*), Rubisco small subunit (*AtRBCS*), chalcone synthase (*AtCHS*) and eukaryotic initiation factor 4a (*AteIF4*) as a loading control. Total, primarily ribosomal RNA (rRNA) is also shown.

**FIGURE 7:**
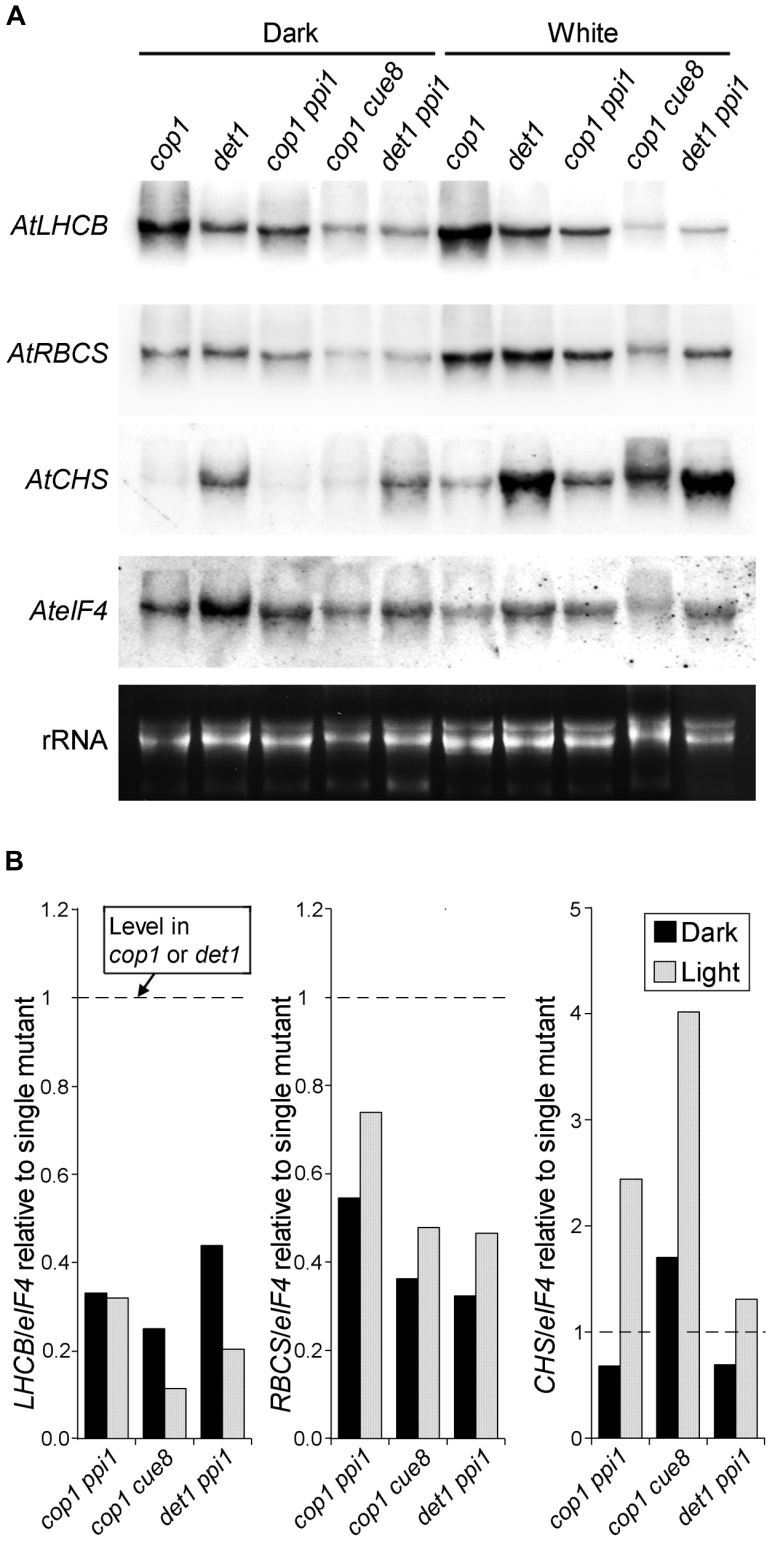
**RNA gel blot analysis showing the genetic interaction between plastid developmental mutations (*ppi1* and *cue8*) and de-etiolated mutations (*cop1* and *det1*) in *Arabidopsis*.** **(A)** Seedlings of the single or double mutants indicated were incubated as in **Figure 6**, followed by RNA extraction and gel blot analysis (probes as in **Figure 6**). **(B)** Relative quantitation of the RNA gel blot in **(A)**, with the level of each transcript, normalized against the *eIF4* loading control, in each double mutant, expressed relative to the normalized level for the same transcript in the corresponding single *cop1* or *det1* mutant.

The data above show that plastid control of *Lhcb* expression occurs in pine and, therefore, is likely to have been functional in the last common ancestor of conifers and angiosperms, before the skotomorphogenesis program suppressed the expression of PhANGs in the dark. Data in **Figure 4**, meanwhile, show that the difference between the skotomorphogenic and the photomorphogenic programs can also be observed in pine, albeit it does not affect *Lhcb* expression. One attractive hypothesis is that the repression of PhANGs like *Lhcb* in the dark evolved through the recruitment of the repressors of photomorphogenesis to repress the ability of PhANG promoters to respond to plastid signals (of a positive nature) in the dark, with this repression being relieved in the light. A prediction of this hypothesis would be that removal of the photomorphogenesis repressors would allow the response to plastid signals to be manifest in full, in the dark as well as in the light; in other words, that removal of the photomorphogenesis repressors would result in angiosperm seedlings showing responses of PhANGs to plastid signals more comparable to those of the conifer, determined by the state of the plastids. We set out to test this hypothesis using genetic tools, both mutations in the *DET1* (Chory et al., 1989) and *COP1* (Deng et al., 1991) genes for repressors of photomorphogenesis, and mutations in plastid biogenesis genes necessary in the dark and the light. As plastid biogenesis mutants we used *ppi1*, defective in TOC33, a component of the plastid protein translocon of the outer chloroplast membrane (Jarvis et al., 1998) and *cue8*, with a mutation in a housekeeping plastid protein causing reduced *LHCB* expression in both dark and light, and affecting both chloroplast and etioplast development (Vinti et al., 2005). The prediction of this scenario would be that mutations in plastid biogenesis genes would reduce or suppress the PhANG gene expression phenotype in the dark caused by those in *DET1* or *COP1*. Seedling phenotypes of the combined mutants generated are shown (**Figure 5**), compared to those of the wild type grown in the absence or presence of norflurazon. A *det1 cue8* double mutant was generated but was severely growth-impaired, had poor seedling survival, and could not be examined for gene expression. Single mutants were grown in the dark and the light. Gene expression results (**Figure 6**) confirmed those previously reported: higher transcript levels were observed in the dark for photo-regulated genes in *det1* and *cop1* relative to those in their wild types; reduced expression of *LHCB* and of a second light and plastid-dependent PhANG, *RBCS* (the gene for the nuclear-encoded small subunit of Rubisco), was seen in *ppi1* and *cue8*, in the dark (mildly for *ppi1*) and the light. The gene expression phenotype of double mutants, compared with that of the single *cop1* or *det1* mutants, is shown in **Figures 7A,B**. Both plastid mutations, particularly *cue8*, caused reductions in the double mutants of the expression of both PhANGs in the dark and the light, when compared with the mRNA levels in the corresponding single *cop1* or *det1* mutant. In general *cop1* appeared to cause a greater derepression of PhANGs in the dark (even though a response to light remained present), and *cue8* a greater suppression in dark and light. Meanwhile the expression of *CHALCONE SYNTHASE* (*CHS*), encoding an enzyme involved in flavonoid biosynthesis, induced by light as a photoprotectant but unrelated to photosynthesis, was strongly elevated in the *det1* mutant, and the presence of the second, plastid-biogenesis mutation caused not a reduction, but a further increase in the light (**Figure 7B**). In summary, the prediction that mutations causing defects in plastid biogenesis would reduce or suppress the effect in the dark of mutations in repressors of photomorphogenesis, specifically for PhANGs, appeared upheld.

### Mutations in Greening-related GOLDEN2-LIKE Transcription Factors Suppress the Action of *det1* Mutations on Photosynthetic Antenna Genes

GOLDEN2-LIKE transcription factors (GLKs) have been shown to drive the expression of a number of greening-related genes, and to mediate the nuclear response to defects in chloroplast protein import. We sought to establish whether the response to plastid-to-nucleus signals, which the data above are consistent with photomorphogenesis repressors suppressing in the dark, is mediated by these transcriptional regulators. To do this we generated a *det1 glk1 glk2* mutant (see **Figure 5**). The prediction was that, as with defects in essential plastid proteins, the expression of greening-related genes would be impaired, in spite of the *det1*mutation. We included in the analysis the combined mutants above, for comparison purposes, and examined two members of two subfamilies of *LHCB*, as well as the *CARBONIC ANHYDRASE 1* (*CA1*) gene, one of the top responders to repression by norflurazon (Koussevitzky et al., 2007), and whose product is involved in the photosynthetic carbon reactions.

**FIGURE 8:**
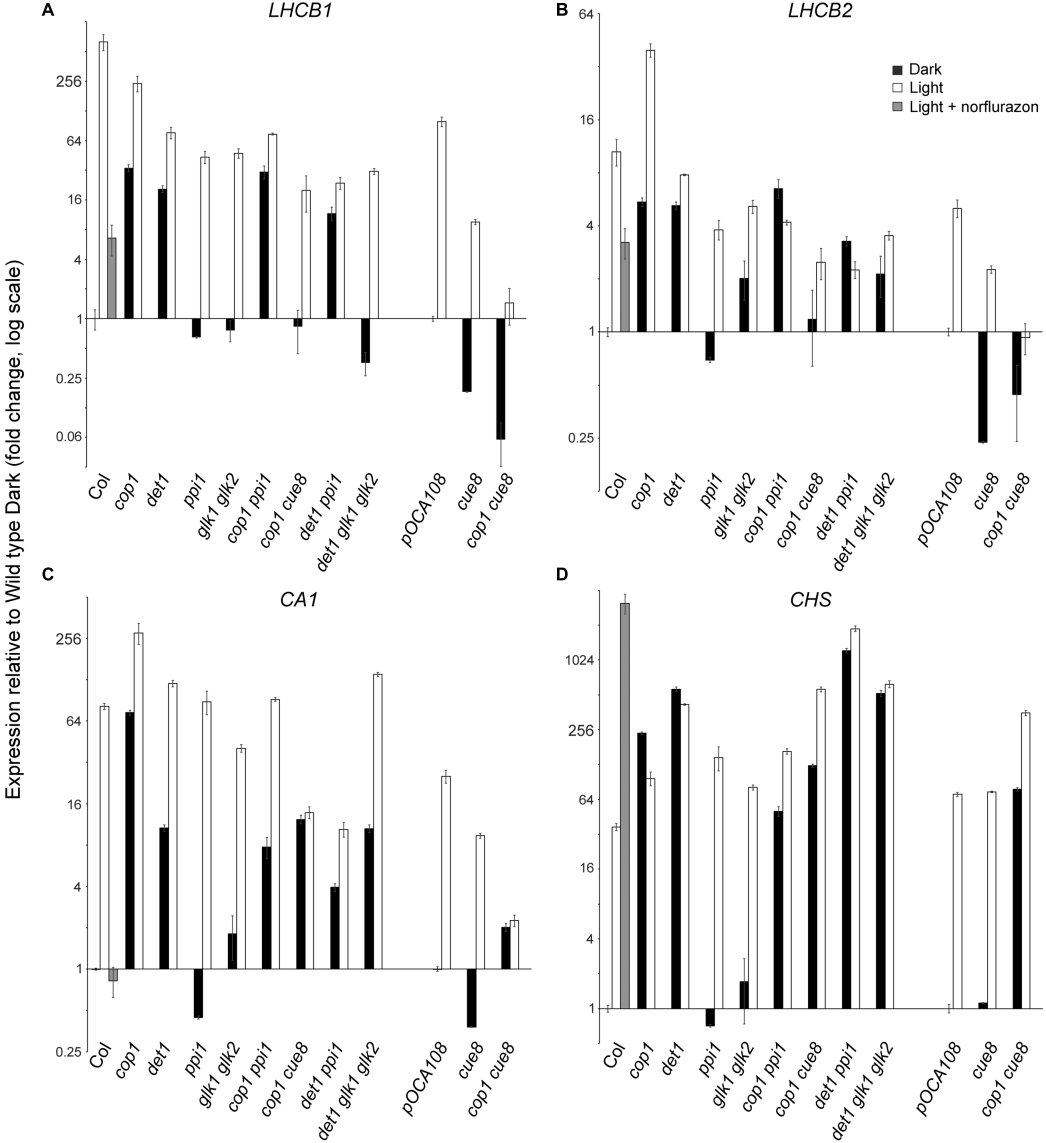
**Quantitative real-time RT-PCR analysis confirming the suppression by plastid developmental mutations of the expression of photosynthesis-associated nuclear genes phenotype in dark of de-etiolated mutations, and showing the requirement of GOLDEN2-LIKE transcription factors for expression of a subset of such genes.** Expression of *LHCB1* **(A)**, *LHCB2* **(B)**, *CARBONIC ANHYDRASE 1* **(C)**, and *CHS* **(D)**, relative to two constitute genes, and expressed in each genotype, on a log scale, normalized against the corresponding expression in the respective wild type. The expression in *cop1 cue8* is shown twice, normalized against each of the wild types.

The results (**Figure 8**) demonstrated the anticipated regulation of *LHCB1*, its expression requiring functional plastids (as shown by the reduction in *ppi* in the light or in *cue8* in dark and light). As expected, repression of *LHCB1* in the dark required the DET1 and COP1 products (note the elevated expression in the mutants), and the impairment in plastid function suppressed the positive effect in the dark of the loss of DET1 or COP1. Loss of GLKs reduced expression of *LHCB1* in the light but also, fully, suppressed the action of the *det1* mutation on this gene, demonstrating that such expression, occurring in the dark if DET1 is absent, necessitates functional GLKs. Nearly identical conclusions could be drawn monitoring the expression of *LHCB2*, albeit the extent of regulation, by both plastid-to-nucleus and light signals, was much attenuated compared to that of *LHCB1* (note also the reduced impact of norflurazon treatment on the wild type). This, however, was not the case for the *CA1* gene, whose expression was light-induced and norflurazon and *CUE8*-dependent, but proved almost completely independent of both the defect in *ppi1* and loss of GLKs. Accordingly, the *glk1 glk2* mutations had no impact on *CA1* expression when combined with loss of *det1*, demonstrating that expression of *CA1*, while suppressed by DET1 in the dark, does not necessitate GLKs. Lastly, expression of *CHS* was, as expected, light-induced, suppressed by DET1 and COP1 in the dark, not reduced by mutations causing plastid defects (if any, elevated), and indeed elevated in the wild type in response to norflurazon. Unsurprisingly, GLK defects had no impact on the expression of this gene.

## Discussion

Our results demonstrate that plastid-to-nucleus communication pathways regulate the expression of the PhANG *Lhcb7* in pine, as well as the activity of its promoter heterologously, in tobacco. Clearly, plastid biogenic signals reporting chloroplast viability either predate the divergence of angiosperms from other seed plants, including conifers, or have evolved repeatedly, the first of those explanations being the more parsimonious. Such divergence occurred at least 140 million years ago, and possibly much earlier (Willis and McElwain, 2014). Evidence exists for plastid-to-nucleus “operational” or environment-dependent signals and even the control of global cellular processes (like cell division) occurring in primitive photosynthetic eukaryotes (Escoubas et al., 1995; von Gromoff et al., 2008; Kobayashi et al., 2009). In *Chlamydomonas*, chlorophyll precursors and their chloroplast export have been shown to mediate the light response of an HSP70 heat-shock protein (Kropat et al., 2000), but the product of this gene is not directly photosynthesis-associated. Also in *Chlamydomonas*, norflurazon was found to cause a loss of chlorophyll but no specific gene expression changes (Johanningmeier, 1988), and changes brought about by tetrapyrrole molecules included very few photosynthesis-associated genes (Voss et al., 2011). Suppression of transcription or translation by very elegant means in this alga does cause reduced expression of some nuclear photosynthesis associated genes, notably *Lhca1* (Ramundo et al., 2013). Hence, the range of plastid function monitored in *Chlamydomonas* is distinct, narrower than that monitored by plastid biogenic signals in flowering plants. This raises the question of whether such regulatory pathways are orthologous. Arguably prior to multicellularity there was little need for the existence of non-photosynthetic proplastids, required to be inherited via germ cells or meristematic plant stem cells, and consequently for the assembly of photosynthetically competent chloroplasts from them as photosynthetic cells differentiated (Andriankaja et al., 2012; Charuvi et al., 2012; Jarvis and López-Juez, 2013). Our evidence, on the other hand, indicates the existence of the classic plastid-to-nucleus ‘biogenic’ signaling pathways controlling chloroplast development outside the angiosperms, and is consistent with such signals evolving or at least expanding in association with the need for differentiation of chloroplast-containing cells from proplastid-containing ones in embryophytes.

**FIGURE 9:**
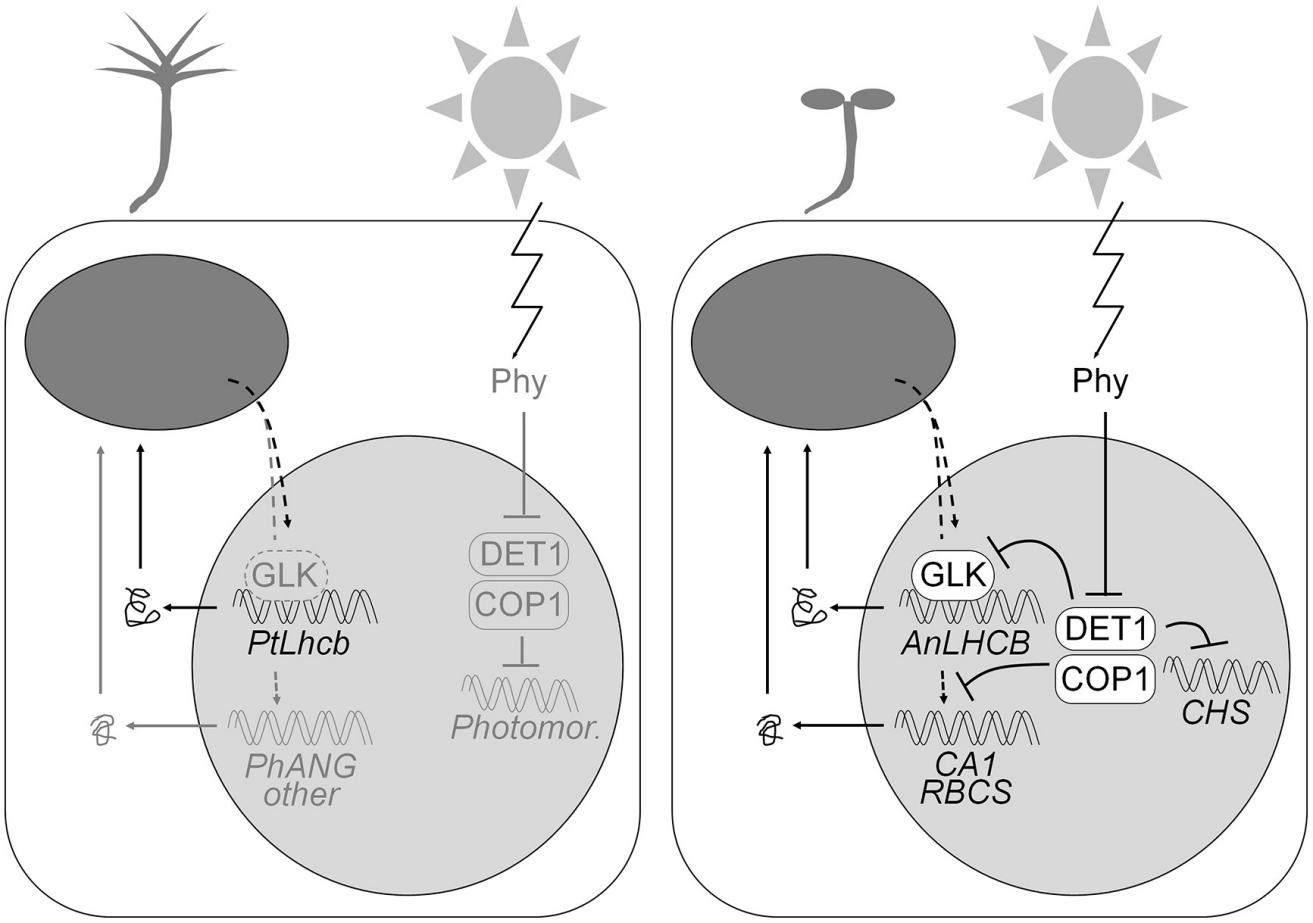
**Model of the regulatory circuitry regulating the gymnosperm (pine) or angiosperm (tobacco or *Arabidopsis*) *LHCB* promoters.** The pine promoter, in either pine itself **(left)** or heterologously expressed in tobacco, is regulated by positive plastid biogenic signals, largely independently of light. The angiosperm *LHCB* promoters, in tobacco or *Arabidopsis* **(right)**, are dependent on positive plastid signals and GLK transcription factors in the dark or the light, but the ability to respond to the plastid signals is suppressed (as shown in *Arabidopsis*) by the DET1 and COP1 proteins in the dark, and so expression only takes place when phytochrome photoreceptors repress DET1 and COP1 and allow the response to plastid signals. Several other photosynthesis-associated gene promoters are regulated in the same fashion but are not dependent on GLKs. DET1 and COP1 regulate the expression of the light-dependent but photosynthesis-unrelated *CHS* gene without the requirement for positive plastid biogenic signals. Components (including general photomorphogenesis genes) known in angiosperms and whose presence is hypothesized in gymnosperms are shown in gray on the left.

Close links between light and plastid responsiveness of PhANG expression have been uncovered at multiple levels. In cereal leaves, which show a basal/apical gradient of chloroplast development, no response of PhANGs to photoreceptors occurs in regions below those in which plastids are transcriptionally active (Rapp and Mullet, 1991). This could be a developmental, rather than a pathway-sharing connection, since a global gene expression analysis of the developing maize leaf has revealed a transition region, before chloroplasts become photosynthetically competent and the tissue converts from sink to source, in which the genes for transcription factors like HY5 and Golden2 become expressed (Li et al., 2010). Nevertheless evidence that light and plastid signals act on common promoter regulatory elements abounds (Bolle et al., 1996; Kusnetsov et al., 1996; Puente et al., 1996; Martínez-Hernández et al., 2002; Gray et al., 2003). One known exception, involving the response element of an *Arabidopsis LHCB* promoter to high light and reactive oxygen (Staneloni et al., 2008), probably reflects the distinction between plastid-to-nucleus biogenic and operational signals. Such overall evidence led to the original proposal (Arguello-Astorga and Herrera-Estrella, 1998) that light-responsive elements may have had an ancestral function as response elements to plastid retrograde (biogenic) signals. We observed a nearly complete lack of response (similar to that in photoreceptor mutants) of the *LHCB* promoter to phytochrome-activating light pulses in plastid-defective *cue8* and *cue3* mutant seedlings, even when some chlorophyll accumulation, and therefore basal *LHCB* expression, was readily detectable (Vinti et al., 2005). Others have also shown the degree of light responsiveness of PhANGs to be greatly attenuated by impairments of chloroplast biogenesis (Ruckle et al., 2007). Our evidence is consistent with the proposal of an ancestral role of light-responsive elements in the response to plastid-to-nucleus signals, and extends it by showing that the action of plastid signals occurs downstream that of the *det1* and *cop1* mutations in the dark, and therefore that the DET1 and COP1 proteins, dark repressors of photomorphogenesis, may repress the action of plastid signals (**Figure 9**). It has been previously shown (Sullivan and Gray, 1999) that lincomycin treatments suppress the elevated *LHCB* expression in the dark of *Arabidopsis cop1* and pea *lip1*. *LIP1* is orthologous to *COP1*. These authors interpreted their data as evidence for plastids producing retrograde signals that repress PhANG expression also in the absence of light. An alternative explanation is that in flowering plants COP1 and DET1 act in the dark to suppress the response to *positive* signals of plastid origin. These plastid-derived signals would not be produced when plastids are inactive (**Figure 9**). Haem, specifically that produced in plastids by the *FERROCHELATASE 1* gene product (Woodson et al., 2011) has been shown to act as precisely such a kind of signal. We should note that placed in the context of earlier data, our model (**Figure 9**) is a plausible one, however, alternative explanations do remain possible, including plastid signaling and photomorphogenesis acting fully independently. Our current evidence cannot rule these out, particularly given that mutations in individual photomorphogenesis repressors, while causing elevation of PhANG expression in the dark, do still allow a light response to be manifest (see **Figures 7** and **8**).

It is important to note that the heterologous pine and native tobacco PhANG promoters respond differently to light in tobacco seedling cells. This implies that any suppression of the response to plastid signals by photomorphogenesis repressors, if occurring, must differentiate between both kinds of promoters. Genetic screens for mild defects in plastid-to-nucleus communication identified mutant alleles of the CRYPTOCHROME 1 photoreceptor and of HY5 (Ruckle et al., 2007) effective even in the absence of light. This led the authors to propose that plastid-to-nucleus signals caused a “rewiring” of light signaling networks, converting HY5 from a positive to a negative regulator of PhANGs, through the plastid-dependent action of a cofactor (Larkin, 2014). The centerpiece of that model is that two interacting factors are co-required for light induction of PhANGs, with one being dependent on plastid function. The evolution of light regulation of individual PhANG promoters may have been based on the fine tuning of such a composite element, and could explain the difference between the two related PhANG promoters in the same cells.

According to our model, the behavior of the pine *Lhcb6* promoter both in its native context and in tobacco seedlings represents the ancestral condition, one in which the expression is independent of repressors of photomorphogenesis, and therefore independent of light (**Figure 9**). Such a model assumes that the repressors of photomorphogenesis predate flowering plants. Indeed COP1 and DET1 are proteins of a wide phylogenetic distribution (Lau and Deng, 2012). Pine seedlings show rudimentary but clear skotomorphogenesis (**Figure 4** and Burgin et al., 1999), indicating not only the presence of photoreceptors, but also of functional photomorphogenic repressors. We identified full or partial homologs of both DET1 and COP1 in a monocot, rice, a lycophyte (sister group to the seed plants and ferns), *Selaginella moellendorffii*, and in the moss *Physcomitrella patens* (**Supplementary Figures S3** and **S4**). This confirms that photomorphogenesis repressors predate angiosperms. Skotomorphogenesis in gymnosperms does not involve repression of PhANG expression, or does so only very mildly (Alosi et al., 1990; Yamamoto et al., 1991; Peer et al., 1996). An exception is *Ginkgo biloba*, a divergent gymnosperm whose seedlings fail to green in the dark; however, this appears due to an alternative mechanism, repression not of photomorphogenesis but of the ability to respond to circadian clock signals (Christensen and Silverthorne, 2001). It is therefore most plausible that the regulatory condition of *LHCB* expression in the last common ancestor of gymnosperms and angiosperms was dependence on plastid signals but independence on light (**Figure 9**).

While the present study was not intended to reveal the sources or nature of plastid-to-nucleus signals themselves, our genetic analysis of the interaction between photomorphogenic repressors and plastid functional state led to some interesting observations. While DET1 acts as a repressor of PhANG expression in the dark, its loss also causes a reduction of expression in the light (**Figures 7** and particularly **8**). This apparent action of DET1 as a repressor in the dark and an activator in the light seems paradoxical, but it has been observed in the past (Chory et al., 1989; Ruckle et al., 2012), and could be related to oxidative damage, which might also explain the large increase in *CHS* (photoprotectant) expression. It was useful to note differences in the responses of the two mutants with dysfunctional plastids, *ppi1* and *cue8*. *ppi1* is defective in TOC33, a component of the receptor complex at the outer envelope for chloroplast protein import specializing in the assembly of photosynthetically competent chloroplasts (Bauer et al., 2000; Kubis et al., 2003). Accordingly, defects in PhANG expression extended to most PhANGs tested (see **Figures 6–8**) but occurred clearly in the light, being mild (**Figures 6** and **7**) or barely, if at all, detectable (**Figure 8**) in the dark. On the other hand, *CUE8* encodes a chloroplast housekeeping protein, defects in which cause altered etioplast and chloroplast development (Vinti et al., 2005). This correlates with defective PhANG expression in both the dark and the light (see, in particular, **Figure 8**). This confirms the capacity of all plastids, not just chloroplasts, to communicate their physiological state to the nucleus, and the need for both housekeeping and photosynthesis-associated protein function to maintain PhANG expression. Our classic inhibitor treatments (norflurazon and lincomycin) also lead to similar conclusions. While lincomycin is a translation inhibitor specifically affecting chloroplast ribosomes (see Gray et al., 2003), regardless of light, norflurazon applied from germination has been shown to prevent functional photosynthetic membrane complex assembly in the light (Voigt et al., 2010; Kim and Apel, 2013). It is worth remembering that tetrapyrrole, including chlorophyll, biosynthesis occurs in association with the inner chloroplast envelope as well as the thylakoids (Tanaka et al., 2011). These inhibitors reveal the broad “base” of plastid physiology (protein synthesis machinery, tetrapyrrole synthesis, membrane state/function) being reported by biogenic plastid signals.

Also of interest is the observation that defects in GLKs did not affect all PhANGs tested. This is consistent with the original, extensive analysis (Waters et al., 2009), which identified as GLK targets primarily the genes for light harvesting, reaction center, and electron transport proteins and for chlorophyll biosynthesis. The authors did not observe defects in, for example, *RBCS*, the nuclear gene for the core protein of the photosynthetic carbon reactions, nor did we observe an effect for *CA1*. GLKs are themselves targets of plastid and light signals (Waters et al., 2009) and they can mediate the action of plastid signals on other genes (Kakizaki et al., 2009, and this study), but they cannot be solely responsible for this action. Interestingly, the response of *LHCB*s in *glk1 glk2* mutants to light was mildly affected, but expression clearly did not occur in such mutants in the dark, even in the absence of DET1, revealing GLKs as possible targets for the dark repression of *LHCB*s by DET1. An alternative explanation, that *glk1 glk2* mutants simply displayed globally impaired chloroplast development, and therefore reduced plastid signaling, is not compatible with the fact that the impact of the mutations was gene-specific. Instead the evidence supports their direct role in the expression in the light of a subset of genes. The normal light response of *glk* mutants and the apparent additive effect (implying independence) of reduced greening between the phenotypes of *glk* and *phytochrome B* mutations observed in a previous study (Waters et al., 2009) could have arisen from a combination of photoreceptor redundancy and the fact that the pale phenotype of *phytochrome B* is due in part to its altered, shade-type, reduced leaf cellular development (Tsukaya, 2005).

Perhaps one unexpected consequence of the present work is that current progress in the understanding of terminal, mechanistic steps in both light and plastid-to-nucleus signal action might lead to mutually revealing findings, and assist an eventual goal of rational engineering of chloroplast biogenesis.

## Author Contributions

AH and EL-J designed research. AH, SK, and EL-J carried out research. AH and SK contributed equally to this work. EL-J wrote the manuscript.

## Conflict of Interest Statement

The authors declare that the research was conducted in the absence of any commercial or financial relationships that could be construed as a potential conflict of interest.

## Footnotes

1 http://www.ncbi.nlm.nih.gov/genbank/

2 http://www.ebi.ac.uk/Tools/msa/

3 http://www.ch.embnet.org/
