# Supplementary material for: Chloroplast Biogenesis-Associated Nuclear Genes: Control by Plastid Signals Evolved Prior to Their Regulation as Part of Photomorphogenesis
Source: Front Plant Sci. 2015 Dec 10;6:1078. doi: 10.3389/fpls.2015.01078 (PMC4674571; doi:10.3389/fpls.2015.01078)
Supplement: Supplementary file 1 [file Presentation_1.PDF]

Figure S1. Quantitation of tobacco RNA gel blots

A Response to light of transcripts in tobacco
(Quantitation of RNA gel blot in Fig. 1)

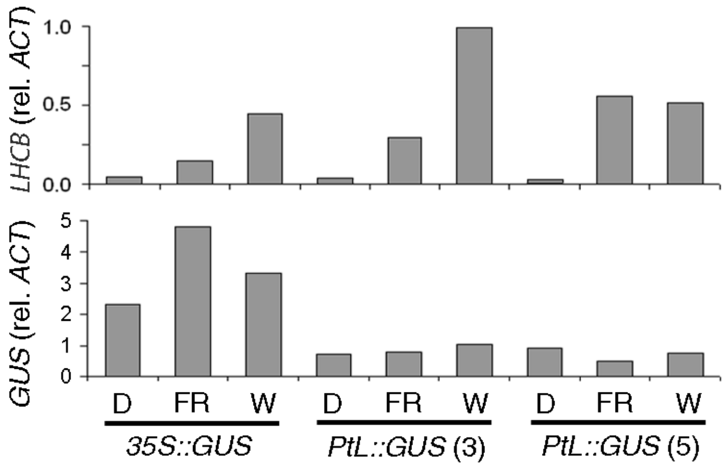

B Response to norflurazon of transcripts in tobacco
(Quantitation of RNA gel blot in Fig. 2A)

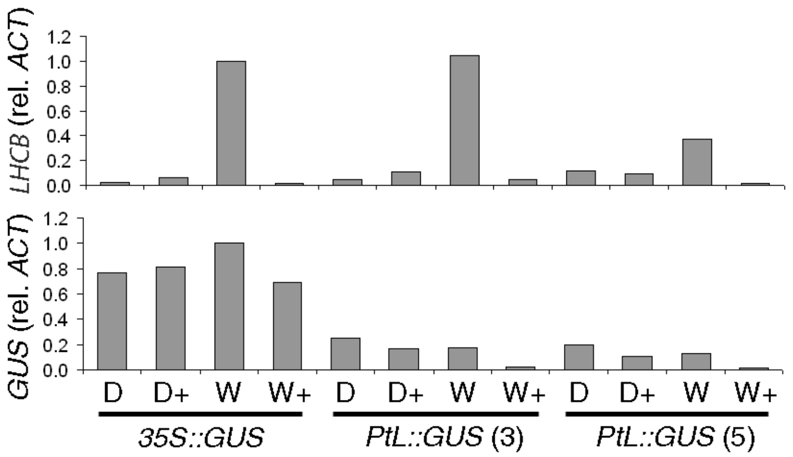

C Response to lincomycin of transcripts in tobacco
(Quantitation of RNA gel blot in Fig. 2B)

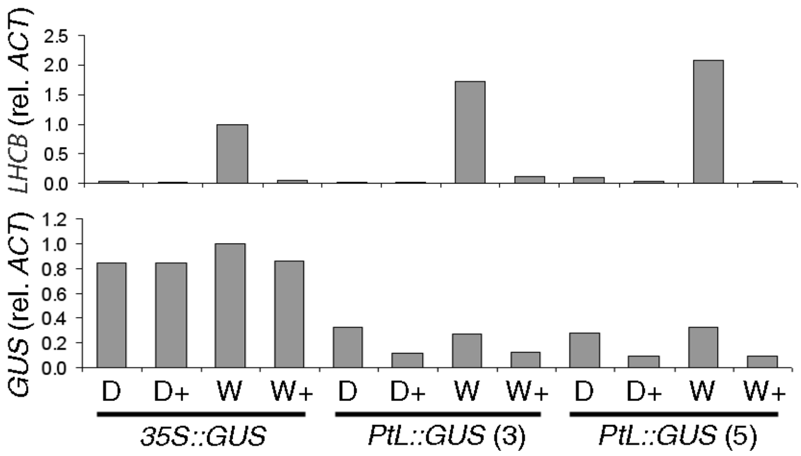

Figure S2

Response to light of *LHCB* in pine
(Quantitation of RNA gel blot in Fig. 4)

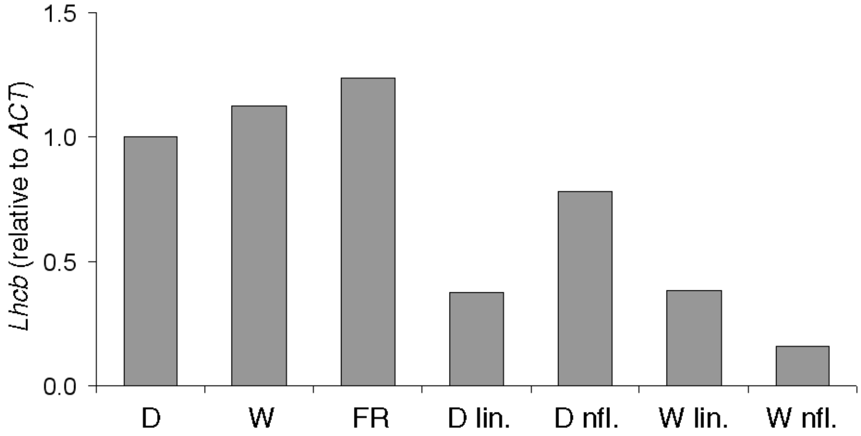

Figure S3. DET1 homologues.

```
At   ---MFTSGNVTARVFERQIRTPPPGASVNRARHFYENLVPSYTLYDVESPDHCFRKFTED
Os   MATFFRSANLASRVFDRQFLSPRPGATVNTVRQFYENLVPSYTICDIDCPDYSFRKFTDD
Pg   ------------------------------------------------------------
Sm   --MVKRSENITHRLFNRQLSCKPSGSQLHTVRTLYENIVPSHTVYDVECPDHTFRKFTND
Pp   --MWKQSENIVHRLFSRQVSSGRPNTTIHRARELYENVVPSHTVYEVDCPDQWWRRFTHD

At   GLFLISFSRNHQELIVYRPSWLTYSTTDDSTTTLPPLPRRASKFDSFFTQLYSVNLASS-
Os   GNYLVAFSRNHQDLIVYRPIWPTFSCNEPCD--SHDLPPKAKKFDSFFKQLYSISLASS-
Pg   ------------------------------------------------------------
Sm   GQYLIAFTRSFQHLVVYRFNWLNFCVKGEAE--DAELPPKAAKFESFFTELYCVALATGT
Pp   GQYLICFSKAQQDLLIYRPLWPTYCSTSDT---INDLPSKSKEFESYFSLLHQVPLARGP

At   NELICKDFFLYHQTRRFGLFATSTAQIHDSSSPSNDAVPGVPSIDKITFVLLRLDDGVVL
Os   NEYICKDFFLYMECHQFGLFATSTAQSNDSS-ATEGAIHGVPSIEKITFYLVRLDDGAIL
Pg   ------------------------------------------------------------
Sm   -ETICKDFFLTTENGVYGLFATSTGPDADAA-SVQGAVPGVPSIEKIRILAVRLADGIIT
Pp   NEVICKDFFLSTENNLYGIFATSTAPDTNAA-ATNGAVPGVPCIEKITFLVVRFADGSIT

At   DERVFLHDFVNLAHNMGVFLYDDLLAILSLRYQRIHLLQIRDSGHLVDARAIGYFCREDD
Os   DEKAFRNDFINLAHSIGAYLYEDLLCIVSLRYQTIHVLQIRDSGNLVEVRKIGAFCQEDD
Pg   ------------------------------------------------------------
Sm   DDRIYHDDFIHLSHNMGVFMYEDLLAILSVRFQCVHILQIRDSGTFVDVRSVGDFCQEDD
Pp   GRSVFKDDYIHLAHNAGVFLHDDLLAVLSIRFQRIHILQVRDGGLFLDVRTIGDFCRDDD

At   ELFLNSSSQAMMSQDKSKQQSLSGSKEDDTGENGLRHSLSQPSGSNSFLSGVKQRLLSFI
Os   ELFLHSHGQA--------------------------------ARGVSFLPGIKQRLLSFI
Pg   -----DQAENGMQQS-----------------------QQIQYGT--FLGGIKQRLLSFI
Sm   ELLVSSQTQVTFPRSLSESNA----------RSGREQSNSSSETGPPVLGGIKQRLLSFI
Pp   ELILNSQAQVLHNLS-----------------------MKLPQACLQMLRGLKQRLLSFI

At   FREIWNEESDNVMRVQSLKKKFYFHFQDYVDLIIWKVQFLDRQHLLIKFGSVDGGVTRSA
Os   FRKTWNEESDQTLRVQHLKKKFYFHFQDYVDLIIWKVQFLDRHHLFIKFGSVDGGVSRST
Pg   FQSIWNNDTDPVPRVQRLKR--YYHFQHYVDLVMWKVQFLDRHHLLIKFGSVDGGMSRTS
Sm   FRSILEHDDDHVQRAQR-LKRFYYHFQHYTELVMWKVQFLDRYHLLIKFGSADGVISRSC
Pp   LQGIHNEVTTPAKA-QQ-LKRFYYYFQDYMNLVMWKVQFLDRYHLLIKFGSVDGVILRNS

At   D--HHPAFFAVYNMETTDIVAFYQNSAEDLYQLFEQFSDHFTVSS-STPFMNFVTSHSNN
Os   E--QNSAFFAVYNMETTDIVSLYQNSSEELYSLFEYFYDHFHTNPQNSSHGNFISSHSNN
Pg   D--YQTSFFAVYNIETTELLAFYQNSSEELFCLLEQYCDQFRAPSQYPLHTRFISSYNNN
Sm   DVSQQLAFFVVYNFETTEILEFQQNSSLELLQLFENFADHFRSAQRYPVYMNYVTSFSNN
Pp   EVSHQNSFFAVYNMETTEILAFYPNSSEELLQLVEHYWDHFRVVPQSPLYMNFISSYSNN

At   VYALEQLKYTK---------NKSNSFSQFVKKMLLSLPFSCQSQSPSPYFDQSLFRFDEK
Os   VHALDQLRTIK---------NKANSTSQFVKKMMASLPYTCQSQSPSPYFDLSLFRYDEK
Pg   IHVREQLRKQKAACIESK----AGSYSQFVKKTLACMPFSSQSQSPSPYFDQSLFHFDEK
Sm   VFVREQSREQKVACINTKASNKANSFAPIARRTLASLPVNSQSQSPSVYFDHSLFHFDEK
Pp   IFAREQLRKQKAACISGK----IGSYAQVIKRTLASLPHNSQSLSPSAYFDQYLFHFDEK

At   LISAADRHRQSSDNPIKFISRRQPQTLKFKIKPGPECGTADGRSKKICSFLFHPHLPLAI
Os   LISAIDRHRHCTEHPIKFISVKQPNVVKFKIKPGSDSGASDSRAKRISSFLFHPFFPLAL
Pg   LISATDRHRQCMEHPIQFKLKRYPNTLKFKINPGLEAGANDGRTKRVASFIFHPIFPFAI
Sm   LISATDRHKPSMEHPIKFLSRRRPHILKFKINPGPENGANETRIKRVAAFVFHPIFPFAI
Pp   LISSTDRHKPCAEHPIKFISRRRPNTLKFKINPGLELGSNDGHIKRVASFLFHPIFPFVI
```

```
At   SIQQTLFMPPSVVNIHFRR
Os   SIQQT-YMQPTVVNLHFRR
Pg   SIQQA-FMQPSVVN-----
Sm   SIQQS-FLQASVVNFHFRK
Pp   SVQQS-FMQTSIVNFHFRR
```

At: *Arabidopsis thaliana* (At4g10180). Os: *Oryza sativa* (Os01g0104600). Pg: *Picea glauca* (partial sequence, UniGene Pgl.17225). SM: *Sellaginella moellendorffii* (SELMODRAFT_104399). Pp: *Physcomitrella patens* (PHYPADRAFT_195255). Light and dark shading indicate similar and identical aminoacids respectively.

Figure S4. COP1 homologues.

```
At   ------MEEISTD---PVVPAVKPDPRTSSVGEGA----NRHENDDGGSGGSEIGAPDLD
Os   --MGDST---VAGALVPSVPKQEQAPSGDASTAALAVAGEGEEDAGARASAGGNGEAAAD
Pg   ------------------------------------------------------------
Sm   -----------------------------------MSFSSE----QQQQQQQQGPRLLAD
Pp   MEGGGPFTQGSAGPHVPSVAEVKQEPRTGSWS-----------SEDNKYEVAMATKPALD

At   KDLLCPICMQIIKDAFLTACGHSFCYMCIITHLRNKSDCPCCSQHLTNNQLYPNFLLDKL
Os   RDLLCPICMAVIKDAFLTACGHSFCYMCIVTHLSHKSDCPCCGNYLTKAQLYPNFLLDKV
Pg   ------------------------------------------------------------
Sm   KDFQCPICIQTMKDAFLTACGHSFCYMCIITHLNNKKNCPCCGVYLTSSQLYPNFLLNKI
Pp   KDFLCPICIQTMKDAFLTACGHSFCYTCIMTHLSNKSNCPCCGLYLTNNQLFPNFLLNKL

At   LKKTSARHVSKTASPLDQFREALQRGCDVSIKEVDNLLTLLAERKRKMEQEEAERNMQIL
Os   LKKMSARQIAKTASPIDQFRYALQQGNDMAVKELDSLMTLIAEKKRHMEQQESETNMQIL
Pg   ------------------------------------------------------------
Sm   LKKVATSQI-SCASPTEQLRFALQQGVDVPLKEIDSLLVLLSDKKRKAEHEEAEVNMEVL
Pp   LGKASASHLVSNASPAENLRLALQQGVDLPVKELDSLMRLLSEKKRKAEQEEAETNMEIL

At   LDFLHCLRKQKVDELNEVQTDLQYIKEDINAVERHRIDLYRARDRYSVKLRMLGDDPSTR
Os   LVFLHCLRKQKLEELNEIQTDLQYIKEDISAVERHRLELYRTKERYSMKLRMLLDEPAAS
Pg   ------------------------------------------------------------
Sm   LEFLQRSRQQKMEELSEIQGDLQSLREDISAVEIQRQELLKSRQTSSLKWRFLLDCPALD
Pp   LEFLHRSRQQKQEELSLLQGDLQFLKEDIITVEKQRQDLLRAKEKYALKIRMIGDGPSTS

At   NAWPHEKNQI---GFNSNSLSIRG-GNFVGNYQNKKVEGKAQGSSHGLPKKDALSGSDS-
Os   KMWP-SPMDKPSGLFPPNSRGPLSTSNPGGLQNKKL-DLKGQISHQGFQRRDVLTCSDPP
Pg   ------------------------------------------------------------
Sm   CAAQENPYNRPAVP-----------LHRVGQAGAALLGGEQRKATRGPLVKKNGGISNGT
Pp   MPD---TLAA----CEKTSKSGVTSQKRGGQGGGVSSGKNQLDSQG--------------

At   QSLNQSTVSMARKKRIHAQFNDLQECYLQKRRQLADQPNSKQE---------NDKSVVRR
Os   SAPIQSGNVIARKRRVQAQFNELQEYYLQRRRTGAQSRRLEE----------RDIVTINK
Pg   ------------------------------------------------------------
Sm   SDFEPLPCKTAKKKRMLNQFEDLQDCYLNKRRRDRQMKKLE-----AIVKKEKDEEGCGG
Pp   LAPSPAVMTMAKKRRVVAQFEDLQEAYLQRRRKVAQVQRQKQNVNEAIVRKDEEVHSAGS

At   EGYSNGLADFQSVLTTFTRYSRLRVIAEIRHGDIFHSANIVSSIEFDRDDELFATAGVSR
Os   EGYHAGLEDFQSVLTTFTRYSRLRVIAELRHGDLFHSANIVSSIEFDRDDELFATAGVSK
Pg   ------------------------------------------------------------
Sm   FNQPSGLEDFRSILAGFTRYSRLELVAELRHGDLFHSSNIVSSIEFDRDDELFATAGVSR
Pp   DRYCSGLNDFQSVLTAFTRYSRLRVIAELRHGDLFHSSNIVSSIEFDRDDQLFATAGVSR

At   CIKVFDFSSVVNEPADMQCPIVEMSTRSKLSCLSWNKHEKNHIASSDYEGIVTVWDVTTR
Os   RIKVFEFSTVVNEPSDVHCPVVEMATRSKLSCLSWNKYSKNVIASSDYEGIVTVWDVQTR
Pg   ------------------------------------------------------------
Sm   RIKIFEFATVVNELADVHCPVAEMSTRSKLSCLSWNKYIKGQIASSDYEGTVTVWDVNSC
Pp   RIKVFEFATVVNELADVHCPVVEMSTRSKLSCLSWNKYINSHIASSDYEGIITVWDVNTH

At   QSLMEYEEHEKRAWSVDFSRTEPSMLVSGSDDCKVKVWCTRQEASVINIDMKANICCVKY
Os   QSVMEYEEHEKRAWSVDFSRTEPSMLVSGSDDCKVKVWCTKQEASAINIDMKANICSVKY
Pg   ------------------------------------------------------------
Sm   QSVMEYEEHERRAWSVDFSRTDPTMLVSGSDDGKVKLWCTRQETSVLNIDMKANICCVKY
Pp   QSIMEYEEHEKRAWSVDFSRTDPTMLVSGSDDGKVKIWCTRQESSVINIDMKANICCVKY
```

```
At   NPGSSNYIAVGSADHHIHYYDLRNISQPLHVFSGHKKAVSYVKFLSNNELASASTDSTLR
Os   NPGSSHYVAVGSADHHIHYFDLRNPSAPVHVFGGHKKAVSYVKFLSTNELASASTDSTLR
Pg   ------------------------------------------------------------
Sm   NPGSSNFVAVGSADHHIHYYDLRNSKSPLHVFSGHRKAVSYVKFVSPNELASASTDSTLR
Pp   NPGSSSYVAVGSADHHIHYFDVRNSHMPLYMFNGHRKAVSYVKFLSPTELASASTDSTLR

At   LWDVKDNLPVRTFRGHTNEKNFVGLTVNSEYLACGSETNEVYVYHKEITRPVTSHRFGSP
Os   LWDVKENCPVRTFRGHKNEKNFVGLSVNNEYIACGSETNEVFVYHKAISKPAANHRFVSS
Pg   LWDVRDNCLVRTFKGHTNEKNFVGLTVNSEYIACGSETNGVFVYHKAISKPAAWHKFGSP
Sm   LWDVQKSSQIRSLTGHANEKNFVGLTVNSEYIACGSETNEVVVYHKEVPKPAARHRFTNH
Pp   LWDVKDNCPTRTLKGHTNEKNFVGLTVNSEYIACGSETNEVFVYHKAMSKPASWHRFGSQ

At   DMDDAEEEAGSYFISAVCWKSDSPTMLTANSQGTIKVLVLAA
Os   DLDDADDDPGSYFISAVCWKSDSPTMLTANSQGTIKVLVLAP
Pg   DLDDSDDDT-SHFISAVCWKSESPTMLAANSQGTIKVLVLAP
Sm   DSEESDDDV-FHFISAVCWKSDSPTMLAANSQGTIKVLVLAP
Pp   DAEESDDDT-SHFISAVCWKSESPTMLAANSQGTIKVLVLAP
```

At: *Arabidopsis thaliana* (At2g32950). Os: *Oryza sativa* (Os02g0771100). Pg: *Picea glauca* (partial sequence, GQ03607_M11). SM: *Sellaginella moellendorffii* (SELMODRAFT_171333). Pp: *Physcomitrella patens* (XP_001771245). Light and dark shading indicate similar and identical aminoacids respectively.

Table S1

Primers used to generate the hybridisation probes for RNA gel blots

Probe	Template type	Template target	Primers
Tobacco *Lhcb*	Genomic DNA	~1.5kb fragment of *Lhcb7* gene from *N. tabacum.* Acc. # X58229	F 5' CCTTCTTTTGCTGGAAATGC 3'<br>R 5' TTACTTTCCGGGGACAAAGTT 3'
Tobacco *ACTIN*	Genomic DNA	~ 600 bp fragment of *AC9* gene from *N. tabacum*. Acc. # X69885	F 5' ATGGTGTCAGCCACACTGTC 3'<br>R 5' AGGGAAGCCAAGATAGAGCC 3'
GUS	Plasmid	1.8kb gene for *uidA* from pBI121 plasmid. Acc. # U12639	F 5' CGTCCTGTAGAAACCCCAAC 3'<br>R 5' ATCCCTTTCTTGTTACCGCC 3'
Pine *Lhcb*	Genomic DNA	~650bp fragment of *Lhcb6* (*cab*-*6*) gene from *P. thunbergii*. Acc. # X61915	F 5' ATTCTGCCGTTTGAAACCAT 3'<br>R 5' CCTGAACGAAGAAACCGAAC 3'
Pine *ACTIN*	Genomic DNA	~250bp fragment of *ACTIN* gene from *P. contorta*. Acc. # - M36171	F 5' GGTTTTGTTCCAGCCATCTC 3'<br>R 5' AACCTCCGATCCAAACACTG 3'

Table S2

Primers used for quantitative, real-time, reverse-transcriptase PCR

Gene	Gene identifier	Primers
*LHCB1*2*	At1g29910	F 5' CCGATCCAGTCAACAACAAC 3'<br>R 5' TCAAACCATCACATACAACCTTC 3'
LHCB2	At2g05100	F 5' TCTGAGCTGAAGGTGAAGGAG 3'<br>R 5' CGTAAGACCAGGCGTTGTTAG 3'
CA1	At3g01500	F 5' GAAGGACTTGTGAAGGGAACA 3'<br>R 5' TTTAACAGAGCTAGTTTCGGAGAG 3'
CHS	At5g13930	F 5' AGAGAAGATGAGGGCGACAC 3'<br>R 5' ACAAGACACCCCACTCCAAC 3'
UBQ10	At4g05320	F 5' GGAGGATGGTCGTACTTTGG 3'<br>R 5' TCCACTTCAAGGGTGATGGT 3'
ACT2	At3g18780	F 5' AAATCACAGCACTTGCACCA 3'<br>R 5' TGAGGGAAGCAAGAATGGAA 3'
